# Acute promyelocytic leukemia with a novel TTMV::RARA fusion initially presenting as vertebral myeloid sarcoma: a case report

**DOI:** 10.3389/fonc.2026.1752011

**Published:** 2026-02-16

**Authors:** Li Ruijia, Zhang Qianqian, Li Xiaohong, Yang Sen, Zhang Nan, Li Jing

**Affiliations:** 1 Graduate School, Hebei University of Chinese Medicine, Shijiazhuang, Hebei, China; 2 Department of Hematology, Hebei Provincial Hospital of Traditional Chinese Medicine, Shijiazhuang, Hebei, China; 3 Second Outpatient Department of Hebei Provincial Government Offices Administration, Shijiazhuang, Hebei, China; 4 Department of Laboratory Medicine, Hebei Provincial Hospital of Traditional Chinese Medicine, Shijiazhuang, Hebei, China

**Keywords:** acute promyelocytic leukemia, myeloid sarcoma, TTMV::RARA, variant APL, whole-transcriptome sequencing

## Abstract

**Background:**

Acute promyelocytic leukemia (APL) caused by the TTMV::RARA fusion gene is extremely rare, with fewer than 10 formally reported cases worldwide, and routine molecular tests often fail to detect it. This case is unique because the disease first manifested as vertebral myeloid sarcoma. Although bone marrow morphology and immunophenotyping strongly suggested APL, routine diagnostic methods could not confirm the disease, and the final diagnosis relied on whole- transcriptome sequencing.

**Case summary:**

A 39- year- old man was admitted for persistent lower back pain and limited movement of the left lower limb. Imaging revealed destruction of the T9 vertebral body with paravertebral and mediastinal soft- tissue masses. Pathology of the resected mass confirmed myeloid sarcoma. The patient developed pancytopenia and coagulopathy. Bone marrow morphology and flow cytometry showed classic features of APL, and all- trans retinoic acid (ATRA) induction therapy was initiated. However, PML:: RARA PCR and RARa FISH were negative, fusion gene screening and karyotyping found no abnormalities, and the diagnosis was revised to AML, prompting a switch to IA chemotherapy. As the diagnosis remained unclear, whole- transcriptome sequencing was performed and revealed a TTMV::RARA fusion, which was confirmed by RT- PCR. The patient was ultimately diagnosed with TTMV::RARA APL. He later discontinued treatment and died months afterward.

**Conclusion:**

This report presents a rare adult case of TTMV::RARA acute promyelocytic leukemia presenting as vertebral myeloid sarcoma. Whole-transcriptome sequencing was essential for diagnosis after routine molecular tests were negative, highlighting the importance of considering rare RARA fusions in APL-like cases lacking PML::RARA. TTMV::RARA APL may be sensitive to ATRA/ATO-based therapy; however, the patient discontinued treatment. Further cases and clinical experience are needed to optimize management strategies for this rare APL subtype.

## 1 Introduction

Acute promyelocytic leukemia (APL) is a distinct subtype of acute myeloid leukemia (AML) typically driven by the PML::RARA fusion gene. Classic APL responds extremely well to treatment with all-trans retinoic acid (ATRA) plus arsenic trioxide (ATO), with excellent outcomes (1). Approximately 5% of APL cases are driven by alternative RARA fusion genes and are classified as variant APL, which shows marked heterogeneity in clinical presentation, therapeutic response, and prognosis (2). In recent years, the extremely rare TTMV::RARA fusion has been sporadically identified; fewer than 10 cases worldwide have been reported, mostly in children and often with extramedullary involvement (3, 4).

Variant APLs are of increasing clinical relevance because standard molecular methods often fail to detect atypical RARA rearrangements, leading to delays in diagnosis and treatment initiation (5). Several alternative fusion partners, such as ZBTB16, NUMA1, and STAT5B, have been documented, each with varying sensitivity to differentiation therapy and chemotherapy, and with heterogeneous clinical outcomes compared with classic PML::RARA APL (6). Importantly, recent evidence suggests that not all variant APLs are resistant to differentiation therapy. For rare fusion events like TTMV::RARA, traditional reverse-transcription PCR (RT-PCR) and fluorescence *in situ* hybridization (FISH) can yield false-negative results due to the unusual genomic architecture arising from viral integration. In such cases, comprehensive genomic approaches—particularly RNA sequencing or whole-transcriptome sequencing—have proven invaluable in uncovering cryptic rearrangements (7).

Because TTMV::RARA arises from a viral insertion event, it is usually undetectable by routine PCR and FISH, making diagnosis challenging. This study reports an adult patient with TTMV::RARA APL who initially presented with vertebral myeloid sarcoma and was ultimately diagnosed only by whole-transcriptome sequencing after multiple routine tests yielded negative results (**Table 1**). Although variant APL has traditionally been considered to have an unfavorable prognosis, emerging data indicate that TTMV::RARA–positive APL may respond to ATRA/ATO–based therapy when appropriately diagnosed and treated (8). Accordingly, early identification of this fusion is critical, as timely initiation or continuation of differentiation therapy may confer a more favorable outcome than previously assumed. This case provides important insights into diagnostic strategies for variant APL.

**Table 1:** Timeline of clinical events, diagnostic workup, and treatments in a patient with TTMV::RARA acute promyelocytic leukemia presenting with vertebral myeloid sarcoma.

Date	Symptoms/events	Diagnostic findings/treatments
2022/4/1	Low back pain with left leg numbness and limited movement	Lumbar spine MRI: disc protrusion, osteophytes
2022/6/17-2022/6/23	Worsening back and leg pain	Thoracic spine MRI/CT: T9 vertebral destruction, paravertebral soft-tissue mass
2022/6/24	Video-assisted thoracoscopic surgery for mediastinal mass	Pathology: myeloid sarcoma, MPO(+), Ki-67 ≈ 60%
2022/7/20	Recurrent back pain, 15 kg weight loss in 6 months	—
2022/7/27	Admission to hematology	WBC 3.04×10^9/L, Hb 79 g/L, PLT 12×10^9/L, D-dimer 5333 ng/mL; bone marrow: 98% abnormal promyelocytes; flow cytometry: CD13+, CD33+, MPO+, HLA-DR–, CD34–
2022/7/30	—	Started ATRA induction therapy
2022/8/1	WBC rise to 13.29×10^9/L, fever, hypoxia	Paused ATRA; hydroxyurea + dexamethasone
2022/8/3	Symptom improvement	Resumed ATRA + ATO dual induction
2022/8/10–2022/8/16	—	PML::RARA PCR negative; RARA FISH negative
2022/8/13	—	IA chemotherapy (idarubicin + cytarabine)
2022/8/20	—	Bone marrow: abnormal cells down to 34%, maturation arrest at myelocyte stage
2022/8/25	—	Whole-transcriptome sequencing: TTMV::RARA fusion detected; RT-PCR confirmation
Follow-up	Patient refused further therapy	Died several months later

## 2 Case description

### 2.1 Clinical presentation

A 39-year-old man with no previous medical conditions presented with persistent dull low back pain and numbness in his left leg following physical exertion in April 2022. He denied hypertension, coronary heart disease, or chronic illnesses, had no transfusion or family history of hematologic disorders, and reported a 20-year smoking history (approximately 20 cigarettes/day) with occasional alcohol use.

Lumbar spine magnetic resonance imaging (MRI) performed at the Third Hospital of Hebei Medical University demonstrated lumbar disc protrusion, osteophyte formation, and mild wedge deformity of the L1 vertebra. Oral medications (details unknown) provided minimal relief, and subsequent intravenous therapy at a local clinic yielded only transient improvement.

**Figure 1:**
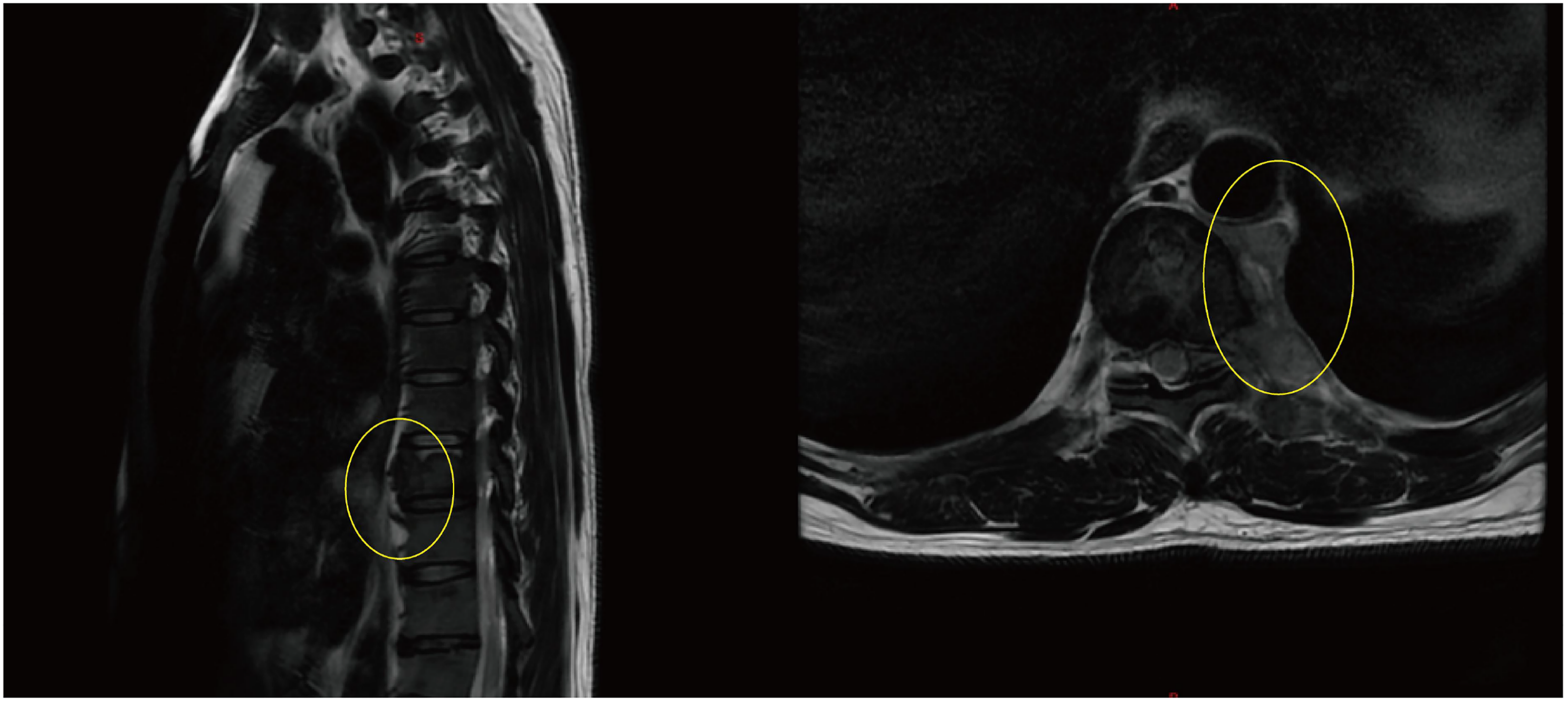
Thoracic spine MRI showing destruction of the T9 vertebral body with adjacent paravertebral soft- tissue mass (The yellow circles indicate the location of the myeloid sarcoma.).

**Figure 2:**
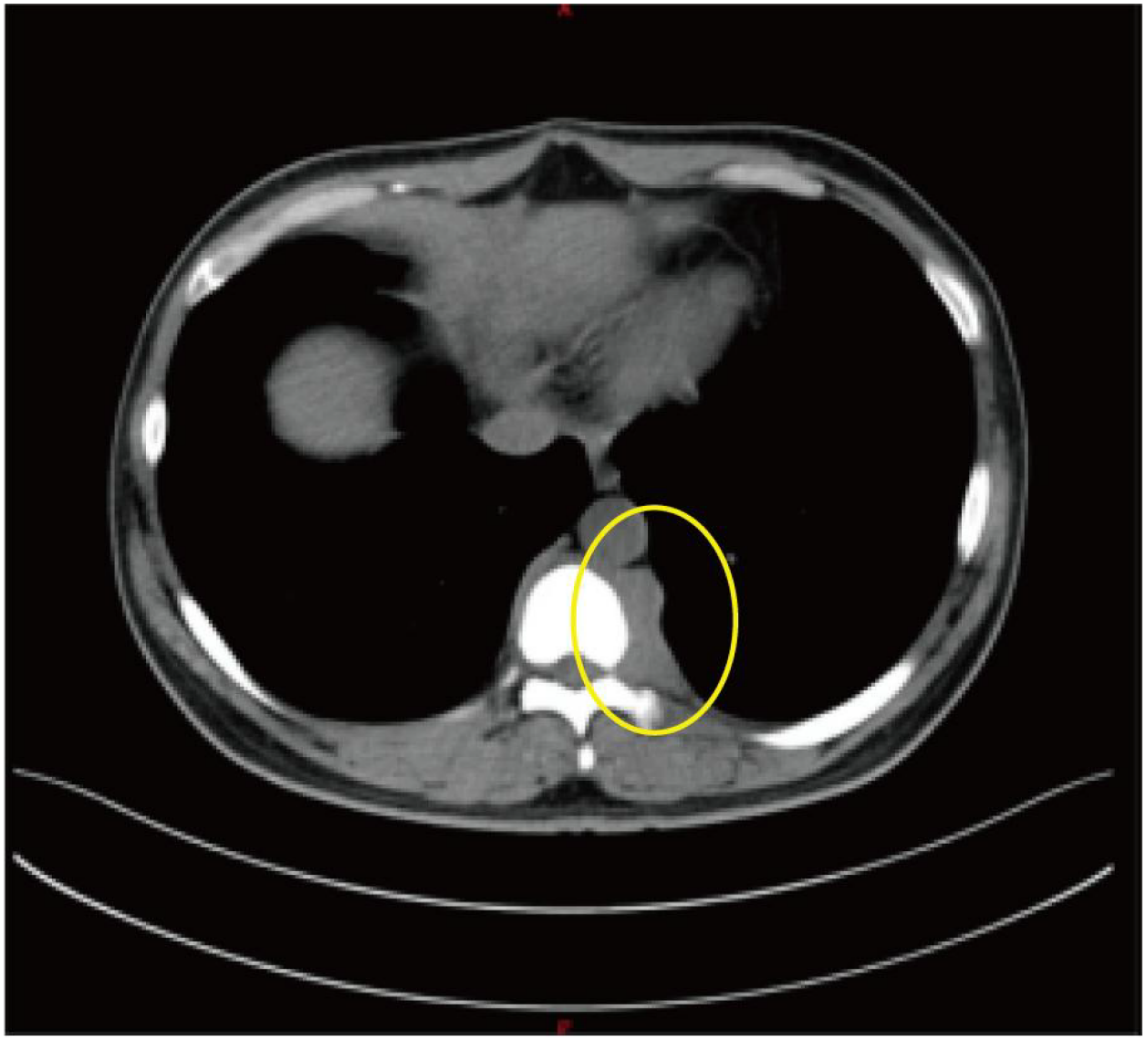
Axial chest CT revealing paravertebral and mediastinal mass (The yellow circles indicate the location of the myeloid sarcoma.).

**Figure 3:**
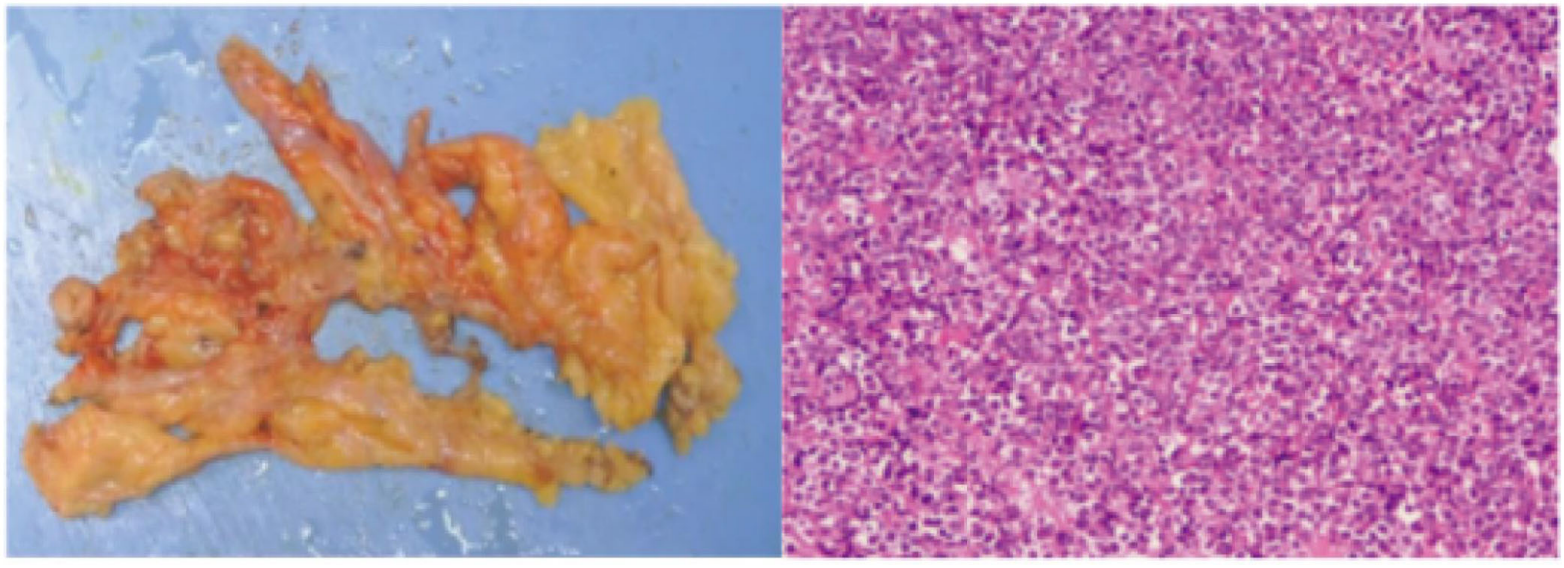
Histopathology of mediastinal mass showing myeloid sarcoma (H&E, ×200).

By mid-June 2022, the patient’s back pain and leg symptoms worsened, prompting admission to the orthopedics department of our hospital. Chest computed tomography (CT) and magnetic resonance imaging (MRI) unexpectedly revealed a mediastinal mass (**Figures 1**, **2**). On June 24, video-assisted thoracoscopic resection of the mass was performed. Postoperative histopathology showed tumor cells positive for myeloperoxidase (MPO) and a Ki-67 proliferation index of approximately 60%. Immunohistochemistry favored a diagnosis of myeloid sarcoma (**Figure 3**).

Approximately one month after surgery, his back and leg pain recurred and progressed, accompanied by a 15-kg weight loss over six months, poor appetite, and sleep disturbance. On July 27, 2022, he was admitted to the hematology department. Physical examination showed a chronically ill appearance, localized spinal tenderness, and mild bilateral lower-extremity edema. Vital signs were: temperature 36.0 °C, pulse 108/min, and blood pressure 151/100 mmHg.

Laboratory findings included WBC 3.04 × 10^9/L, hemoglobin 79 g/L, and platelets 12 × 10^9/L. Peripheral smear exhibited immature cells and marked thrombocytopenia. Coagulation testing indicated a hypercoagulable state with D-dimer 5333 ng/mL and elevated fibrin/fibrinogen degradation products (FDP). Lactate dehydrogenase (LDH) was 1806.6 U/L, and serum ferritin was substantially increased (**Table 2**).

**Table 2:** Key laboratory and diagnostic findings during hospitalization.

Date	WBC ×10^9/L (3.5-9.5)	Hb g/L (130–175)	PLT ×10^9/L (125-350)	D-dimer ng/mL (≤243)	Bone marrow morphology	Flow cytometry	Molecular testing
2022/7/27	3.04	79	12	5333	98% abnormal promyelocytes	CD13+, CD33+, MPO+, HLA-DR–, CD34–	PML::RARA neg
2022/8/1	13.29	81	65	—	—	—	—
2022/8/6	14.24	76	69	—	—	—	—
2022/8/20	—	—	—	—	34% abnormal cells	Maturation arrest	—
2022/8/25	—	—	—	—	—	—	TTMV::RARA fusion (+)

### 2.2 Methods

**Figure 4:**
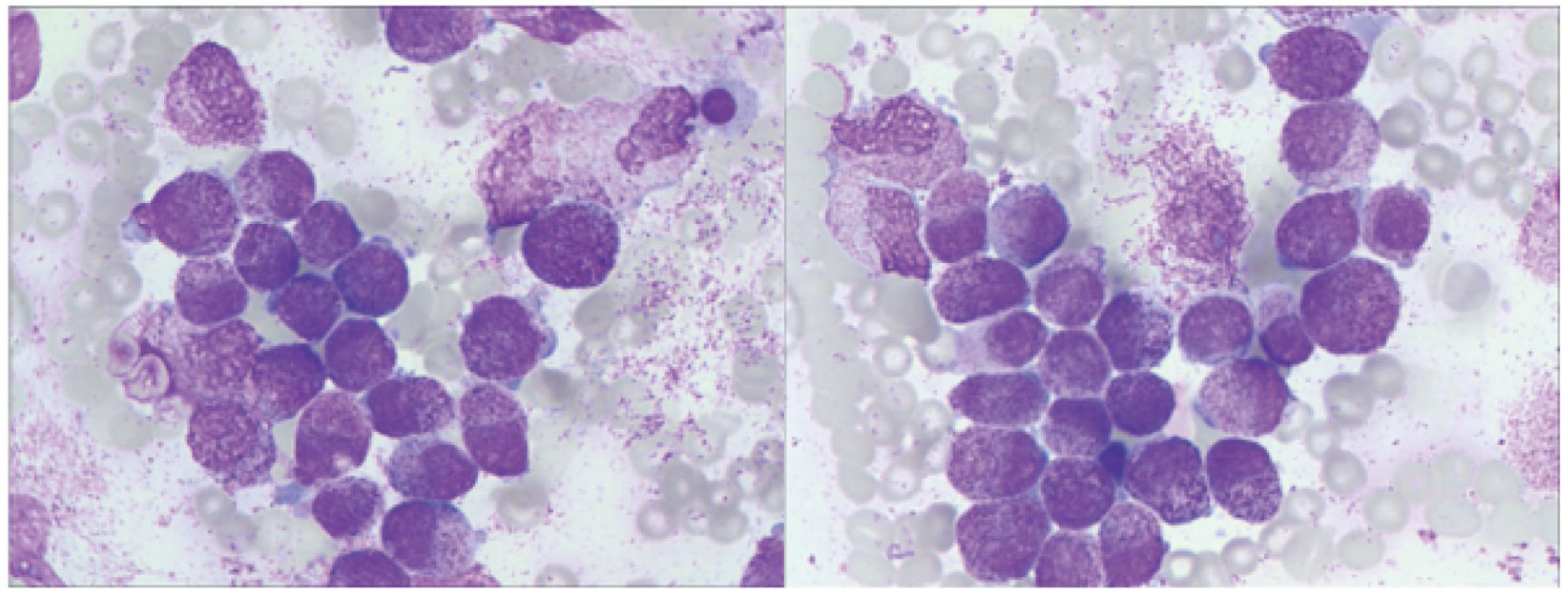
Bone marrow smear showing abnormal promyelocytes with abundant coarse azurophilic granules (Wright- Giemsa stain, ×1000).

Bone marrow aspiration performed on July 27 revealed hypercellularity (nucleated cell ratio ~80%), a granulocyte–erythroid ratio of 65:1, and abnormal promyelocytes accounting for 98% of nucleated cells. The cytoplasm was packed with coarse reddish-purple granules, occasionally containing both primary and secondary granules (**Figure 4**).

**Figure 5:**
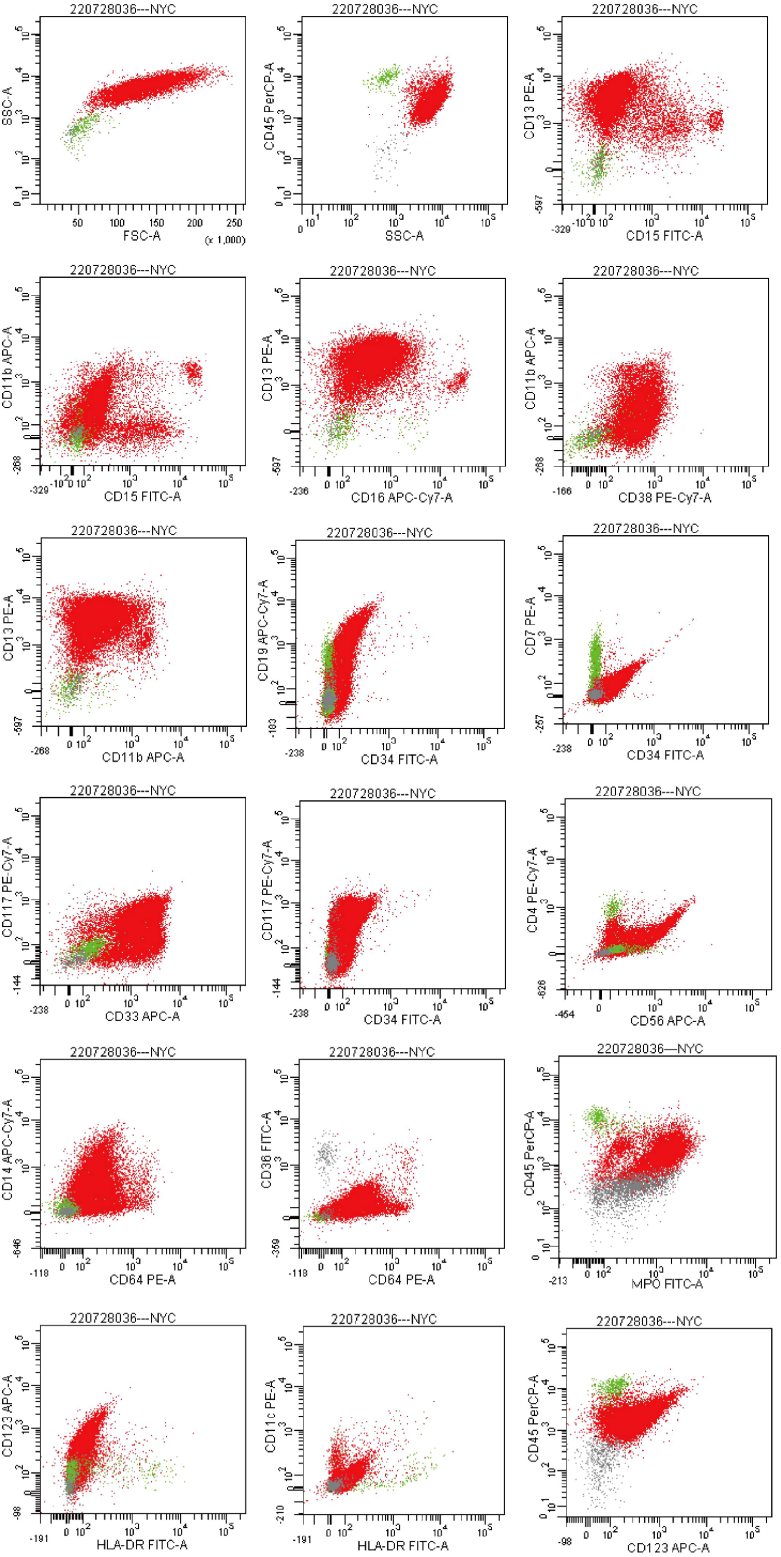
Flow cytometry plots. (Flow cytometric analysis gated on the CD45/SSC dot plot revealed an abnormal cell population with dim CD45 expression and high side scatter, accounting for approximately 96.2% of nucleated cells. The abnormal population expressed CD13, CD33, CD38, CD64, and MPO; a subset of cells expressed CD117 and CD123. The population was negative for HLA- DR and CD34.).

Flow cytometry analysis on July 29 showed that abnormal cells comprised 96.2% of total nucleated cells, expressing CD13, CD33, MPO, CD38, CD64, with partial expression of CD117 and CD123, and negative for HLA-DR and CD34—an immunophenotype highly suggestive of acute promyelocytic leukemia (APL) (**Figure 5**). Bone marrow biopsy findings were consistent with this interpretation.

Differential Diagnosis, Investigations and Treatment:

Considering the morphologic and immunophenotypic features together with significant coagulopathy, APL was strongly suspected. All-trans retinoic acid (ATRA) induction therapy was initiated on July 30. On the third day of ATRA therapy, the WBC count rose to 13.29 × 10^9/L, accompanied by fever and hypoxemia. Differentiation syndrome was suspected; ATRA was temporarily discontinued, and hydroxyurea plus dexamethasone were administered.

After clinical stabilization, ATRA was resumed on August 3, combined with arsenic trioxide (ATO) dual induction therapy.

However, subsequent molecular analysis revealed unexpected results. PCR testing for the PML::RARA fusion gene and FISH analysis for RARA rearrangement were both negative. Comprehensive screening for leukemia-associated fusion genes and karyotype analysis showed no abnormalities, except for detection of a heterozygous DNMT3A frameshift mutation by next-generation sequencing. As these molecular findings did not support classical APL, the diagnosis was revised to acute myeloid leukemia (AML), and idarubicin plus cytarabine (IA regimen) chemotherapy was started on August 13.

Follow-up bone marrow examination after chemotherapy demonstrated a reduction of abnormal immature granulocytes to approximately 34%, but the therapeutic response was unsatisfactory. Flow cytometry indicated granulocytic maturation arrest at the myelocyte stage.

**Figure 6:**
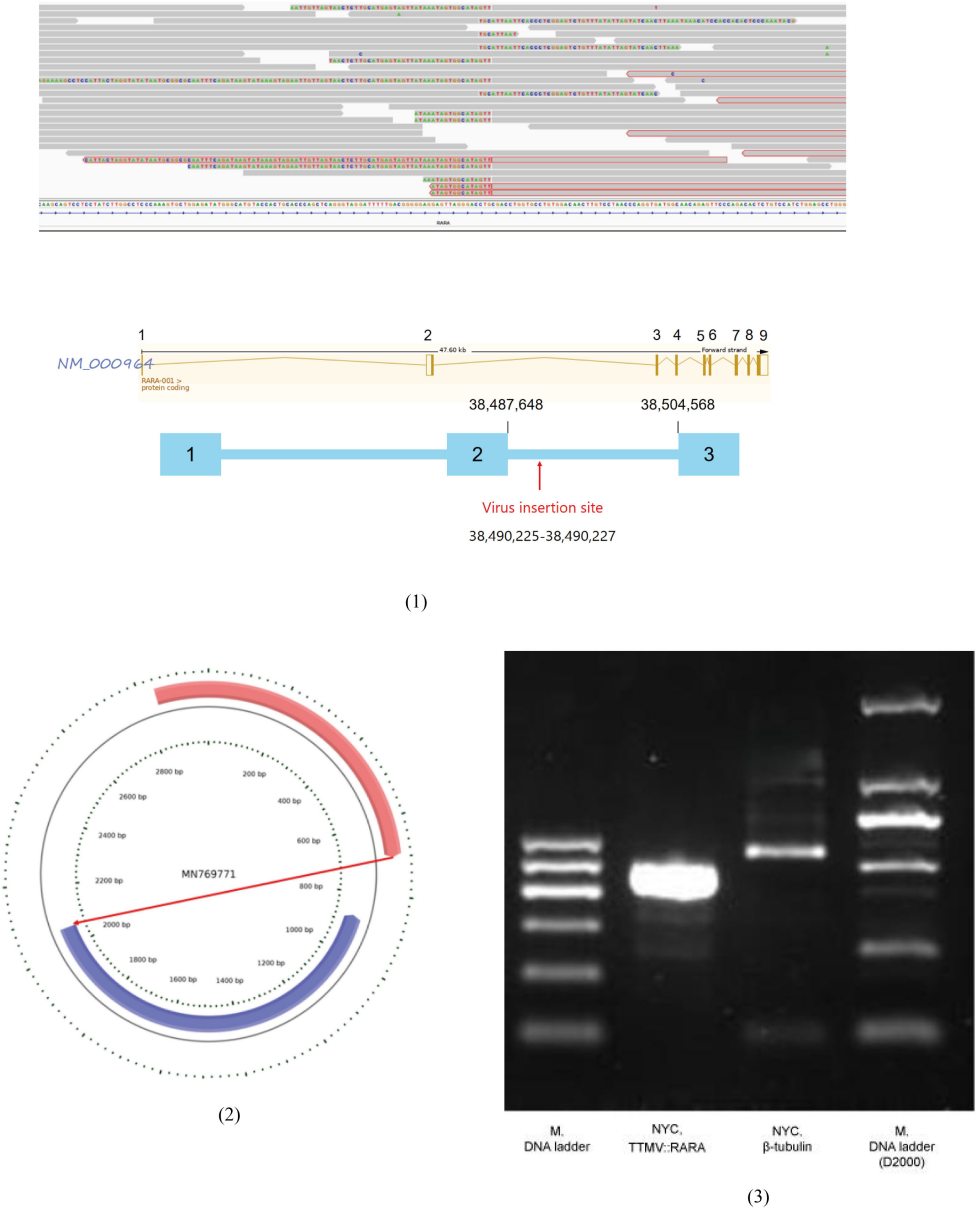
Whole transcriptome sequencing identifying TTMV integration in the RARA gene, confirmed by RT- PCR. (1)A non-human, unknown sequence was found inserted into intron 2 of the RARA gene; (2)Bioinformatic analysis generated a continuous contig of the exogenous insertion sequence with a total length of 2008 nt. Sequence alignment showed high homology with the TTMV strain MN769771.1, but it was divided into two segments: 751 nt (pink) in forward orientation and 1257 nt (blue) in reverse orientation; (3)RNA was extracted from bone marrow smears and subjected to amplification using primers targeting the TTMV::RARA fusion transcript. The expected TTMV::RARA amplicon was obtained and verified by Sanger sequencing.

Given persistent diagnostic ambiguity, multiple multidisciplinary consultations were convened. Bone marrow samples were sent to Lu Daopei Hospital for whole-transcriptome sequencing (WTS). At the RNA level, viral integration sequences were detected. Bioinformatic analysis confirmed Torque teno mini virus (TTMV) insertion into the RARA gene, forming a TTMV::RARA fusion. RT-PCR amplification validated this finding, leading to the definitive diagnosis of TTMV::RARA-positive APL (**Figure 6**).

### 2.3 Results

The confirmed diagnosis was TTMV::RARA fusion-positive acute promyelocytic leukemia, a rare molecular subtype. This case displayed distinct features including extramedullary involvement (vertebral and mediastinal myeloid sarcomas) and typical APL morphology and immunophenotype, despite repeated negative conventional molecular assays. The final diagnosis was achieved only through RNA-based comprehensive sequencing, emphasizing the value of advanced genomic technologies for detecting cryptic RARA fusions.

### 2.4 Prognosis and follow-up

Tragically, by the time of definitive diagnosis, the patient had lost confidence in further treatment and elected to discontinue therapy. Follow-up revealed that he died several months later.

This case highlights the diagnostic challenges posed by variant APLs and underscores the importance of high-throughput sequencing in identifying rare RARA fusion events, such as TTMV::RARA, especially in patients with APL-like morphology but negative conventional molecular results.

## 3 Discussion

### 3.1 Molecular mechanism and clinical features of TTMV::RARA APL

The TTMV::RARA fusion gene is a newly identified molecular subtype of APL, with fewer than 10 cases reported to date (3, 4). TTMV (Torque teno mini virus) is a widely distributed human commensal virus. The mechanism of TTMV::RARA fusion involves insertion of TTMV into intron 2 of the RARA gene, resulting in transcription and translation of a viral (TTMV)–human (RARA) fusion protein (3). This fusion protein retains the ligand- binding domain (LBD) of RARA, but its 5′ end is replaced by viral sequences, disrupting the normal function of RARA. Consequently, myeloid differentiation is blocked and proliferation promoted, ultimately leading to leukemia development (3, 4).

The clinical presentation of this patient shared several similarities with previously reported TTMV::RARA APL cases, including a marked tendency toward extramedullary infiltration. This patient initially presented with myeloid sarcomas involving the vertebrae and mediastinum, which is uncommon in APL. Myeloid sarcoma is a solid tumor formed by infiltrating immature myeloid cells in extramedullary tissues; it is often associated with AML and may occur before, during, or after AML diagnosis (9). In this case, myeloid sarcoma appeared when the complete blood count showed only mild anemia and the peripheral smear was unremarkable, indicating that extramedullary disease was already present at an early stage and thus increased the difficulty of initial diagnosis.

### 3.2 Diagnostic challenges and implications

The diagnostic course of this case exemplifies the difficulties encountered in “APL- like” leukemia when routine diagnostic tests are limited. The patient’s bone marrow morphology (numerous abnormal promyelocytes) and flow cytometric immunophenotype (dim CD45, CD13+, CD33+, MPO+, HLA- DR−, CD34−) were highly consistent with APL, accompanied by a hypercoagulable state (markedly elevated D- dimer), all strongly suggesting APL. However, standard PML:: RARA fusion gene detection (PCR) and RARA rearrangement FISH testing were negative, preventing a definitive diagnosis. Although sequencing detected a DNMT3A mutation, indicating the presence of a myeloid malignancy commonly associated with adverse risk in AML (10), this still could not account for the classic morphological features of APL, leading clinicians to revise the diagnosis to AML and modify the treatment plan and to discontinue ATRA/ATO therapy.

This process highlights the limitations of conventional molecular testing. Current clinical testing for PML:: RARA primarily targets known breakpoint regions or common fusion types. For extremely rare fusions such as TTMV::RARA, produced by viral integration, routine targeted PCR or FISH methods often fail to detect the rearrangement. In this case, only high- throughput whole- transcriptome sequencing successfully identified and confirmed the fusion gene (11, 12). This suggests that for patients with morphology and flow cytometry highly suggestive of APL but with negative routine molecular tests, particularly those with extramedullary involvement, clinicians should be alert to the possibility of variant RARA fusion genes and consider comprehensive molecular testing such as whole- transcriptome sequencing to avoid missed diagnoses and inappropriate treatment interruption.

### 3.3 Treatment response and prognosis

During early ATRA treatment, the patient developed leukocytosis and differentiation syndrome–like manifestations, indicating some degree of differentiation response. Notably, recent evidence indicates that TTMV::RARA–positive APL may be responsive to ATRA/ATO–based therapy. Xu reported that patients harboring TTMV::RARA achieved clinical and molecular responses with ATRA/ATO, suggesting preserved sensitivity to differentiation therapy when appropriately treated (8). In the present case, ATRA/ATO therapy was initiated but discontinued once the diagnosis was revised to AML, which likely contributed to the unfavorable outcome. Therefore, the poor clinical course observed here should not be interpreted as definitive evidence of intrinsically adverse prognosis associated with TTMV::RARA APL. Taken together, these findings suggest that TTMV::RARA APL may not inevitably confer a poor prognosis and that timely recognition and continuation of ATRA/ATO–based therapy may result in more favorable outcomes. Future research is needed to explore optimized treatment strategies for such patients, including intensified induction chemotherapy, targeted therapy, or hematopoietic stem cell transplantation.

### 3.4 Practical implications for laboratory medicine

This case also underscores the critical role of the laboratory in diagnosing difficult hematologic malignancies. When morphology reveals “critical value”- level abnormal promyelocytes, laboratory physicians must promptly communicate with clinicians and initiate rapid diagnostic pathways. At the same time, laboratory professionals should recognize that morphology is not the gold standard and should collaborate actively in performing stepwise molecular testing. When routine tests yield negative results, more comprehensive high- throughput sequencing should be recommended to assist in definitive diagnosis and therapeutic decision- making. Given the rarity of TTMV::RARA, its incidence may be underestimated, prompting consideration of how to develop optimized bioinformatic strategies and detection methods to improve early detection rates of this fusion and to ensure timely initiation or continuation of potentially effective differentiation therapy.

## 4 Conclusion

This report describes a rare adult case of TTMV::RARA acute promyelocytic leukemia presenting as vertebral myeloid sarcoma. The diagnosis was challenging and could only be established by whole-transcriptome sequencing after routine molecular tests yielded negative results, underscoring the importance of considering rare RARA fusions in APL-like cases lacking PML::RARA. RNA sequencing is indispensable for the accurate diagnosis of variant APL. Available evidence suggests that TTMV::RARA APL may exhibit early sensitivity to ATRA/ATO-based differentiation therapy. Unfortunately, the patient discontinued treatment. Accumulation of additional cases and clinical experience is required to better define optimal therapeutic strategies and improve outcomes for patients with this rare APL subtype.

## Data Availability

The raw data supporting the conclusions of this article will be made available by the authors, without undue reservation.
